# Factors Associated with Dental Pain in Mexican Schoolchildren Aged 6 to 12 Years

**DOI:** 10.1155/2017/7431301

**Published:** 2017-06-08

**Authors:** Mauricio Escoffié-Ramirez, Leticia Ávila-Burgos, Elena Saraí Baena-Santillan, Fernando Aguilar-Ayala, Edith Lara-Carrillo, Mirna Minaya-Sánchez, Martha Mendoza-Rodríguez, María de Lourdes Márquez-Corona, Carlo Eduardo Medina-Solís

**Affiliations:** ^1^Faculty of Dentistry, Autonomous University of Yucatan, Merida, YUC, Mexico; ^2^Health Systems Research Centre, National Institute of Public Health, Cuernavaca, MOR, Mexico; ^3^Academic Area of Dentistry of Health Sciences Institute, Autonomous University of Hidalgo State, Pachuca, HGO, Mexico; ^4^Advanced Studies and Research Center in Dentistry “Dr. Keisaburo Miyata”, School of Dentistry, Universidad Autónoma del Estado de México, Toluca, MEX, Mexico; ^5^Faculty of Dentistry, Autonomous University of Campeche, Campeche, CAM, Mexico

## Abstract

**Objective:**

To identify dental pain prevalence and associated factors in Mexican schoolchildren.

**Methods:**

This cross-sectional study included 1,404 schoolchildren aged 6 to 12 years from public schools in the city of Pachuca de Soto, Hidalgo, Mexico. Data were collected through a questionnaire that addressed sociodemographic and socioeconomic factors, eating and dental hygiene habits, and behavior variables. The dependent variable was self-reported dental pain in the 12 months prior to the survey. Data were analyzed using nonparametric statistics and a binary logistical regression model.

**Results:**

Dental pain prevalence among the studied children was 49.9%. The variables associated in the final model (*p* < 0.05) were younger mother's age, higher socioeconomic level, absence of an automobile in the home, fried food, fruit intake, lower tooth brushing frequency, never having used mouthwash or not knowing about it, and parents/guardians with regular to high levels of knowledge about oral health and a regular or good/very good perception of their child's oral health.

**Conclusions:**

One in two children in the study had experienced dental pain in the twelve months prior to the survey. The association of socioeconomic variables with dental pain suggested inequalities among the children in terms of oral health.

## 1. Introduction

Oral disorders such as dental caries and periodontal disease are worldwide public health problems. The “Global Burden of Oral Conditions in 1990–2010” report showed that oral conditions continue to be highly prevalent, affecting about 3.69 billion people. In this report, untreated dental caries in the permanent dentition was identified as the most common of all the evaluated disorders, having the highest worldwide disease load, affecting 35% of all age groups. Severe periodontitis was the sixth most prevalent condition (11% of world population) and untreated caries of the primary dentition was the tenth (9% of world population). Severe tooth loss was the 36th most prevalent condition, affecting 2% of the world's population [1–4]. A number of studies from around the world state that 60–90% of schoolchildren suffer from dental caries [5]. In Latin America, dental caries is among the most frequent untreated health conditions among preschoolers, schoolchildren, and adolescents, who have limited access to restorative dental treatment. In Mexico, dental caries is the primary public oral health problem; for example, at 12 years of age between 70 and 85% of schoolchildren exhibit caries in the permanent dentition, with a high prevalence of untreated cases. Dental caries is also the main cause of dental death in various age groups, presenting a serious challenge to the oral healthcare system due to high care costs [6].

Oral and dental diseases can cause pain, suffering, functional deterioration, and diminished quality of life. The high cost of treatment constitutes a substantial burden to the national health system and for individual households. Families often opt to pay directly for dental care in an effort to maintain adequate oral health among household members. In developed countries, oral health treatment represents from 5 to 10% of health expenses. This treatment is unavailable or extremely limited in many developing countries, where affected teeth are often not treated or merely extracted, making them the main source of pain [7–9]. For decision-makers in different countries, especially in the “developing" ones where the burden of disease is high, it is necessary to consider oral health as a priority and having recent epidemiological data is essential.

The experience of pain, considered a normal consequence of organ or system disorders, is a ubiquitous public health concern. Untreated dental caries generally leads to dental pain. Although dental pain can seriously affect peoples' daily life, negatively affecting quality of life, few epidemiological studies on oral health include questions on dental pain. Orofacial pain, and especially dental pain, can cause sleep loss, diminished work effectiveness or academic performance, absence from school or work, weight loss, and avoidance of certain foods. Some researchers treat it as a predictor of dental health service use (usually curative or emergency) [10, 11]. In this type of care, teeth receive nonregressive treatments that can predispose them to loss over time; it can also raise the probability of an edentulous old age [12, 13].

Health needs can be identified through either subjective self-reporting of symptoms, diseases, injuries, and disabilities or a normative method applied by trained health personnel in a health clinic [8, 14]. Indicators based on self-reported health perception have been shown to be good predictors of oral health. An additional advantage is that data for these indicators can be collected from large groups, along with data for other indicators in population groups. This facilitates correlation of health variables with other variables of interest, such as socioeconomic level, sociodemographic aspects, residence, oral health habits, and education level [15, 16]. Self-reported dental and orofacial pain are good oral health indicators because they are related to the presence of dental diseases, such as caries, periodontal disease, and temporomandibular joint disorders. Among children, odontogenic pain prevalence ranges from 5 to 33% [17] and is frequently related to carious injury on the surface of one or more teeth [18]. Dental pain can also be used by dentists to make decisions; for example, in schoolchildren it can be a symptom of the seriousness of the carious injury. Dental pain has even been used to explore the impact of pain on the psychosocial wellbeing of the child patient and the parents [19]. Schoolchildren can experience pain from caries in primary and permanent teeth. Due to lack of awareness, however, parents usually associate dental pain with primary teeth, thinking that once a tooth exfoliates the pain will disappear. This highlights the need for oral health maintenance strategies, including better information on dental caries prevention, to reduce the risk of disorders [19].

Most orofacial pain is due to dental disorders, and acute pain is generally caused by oral conditions, particularly dental caries and periodontitis. However, pathological processes are not necessarily the sole or sufficient cause of this kind of pain. Pain perception can be modulated by cognitive factors such as knowledge, beliefs, and expectations, which in turn can be influenced by the social, economic, and cultural environment of affected individuals. Here we aimed to identify the factors associated with dental pain as an oral health indicator in schoolchildren, aged 6 to 12 years in the state of Hidalgo, Mexico.

## 2. Materials and Methods

### 2.1. Study Design and Sample Selection

This cross-sectional study was focused on schoolchildren attending primary schools in the city of Pachuca de Soto, Hidalgo. Previously published portions of the methodology explain the use of oral health assistants [20] and oral health services at some time in the past [21]. Study design and implementation met WHO recommendations for oral health epidemiological studies [22]. Sample size was calculated based on a smallest estimated proportion (prevalence) of 35%; a 95% confidence level; 3% accuracy; and a 10% no answer rate. The estimated sample population was 1,554 schoolchildren. In the first stage, a random selection was made of 15 of the 93 public primary schools in the city. In the second stage, a random sample was taken from these schools' enrollment lists to choose potential study participants. The chosen students' parents/guardians were invited to participate in the study and study objectives explained to them. A questionnaire was given to those who accepted, and, after reading, they were asked to sign an informed consent form. Reminders were sent to the parents/guardians who had accepted to participate every 7 days after they were given the questionnaire; they were reminded a maximum of three times. The response rate was 73.8% (*n* = 1,158) after the first, 87.8% (*n* = 1,376) after the second, and 93.8% (*n* = 1,470) after the third reminder. Inclusion criteria were (a) enrollment in one of the primary schools in the study and (b) age between 6 and 12 years. Exclusion criteria were (a) parent report of a disease that could affect child oral health and/or (b) parent/guardian not signing informed consent form. Final sample size was 1,404 schoolchildren.

### 2.2. Data Collection and Variables

Data were collected through a questionnaire answered at home by the schoolchildren's parent/guardian. The questionnaire was divided into sections that allowed the collection of sociodemographic, socioeconomic, food habits, oral health habits, oral appearance satisfaction, and oral health services use data. Questionnaires were distributed and recovered through the schools. The schoolchildren's self-report of dental pain was the studied variable. This was measured using the question “*In the last twelve months, has your child experienced any pain/discomfort in the mouth, teeth or gums?*” Results were measured with a dichotomous scale: 0 = no and 1 = yes.

A total of eight independent sociodemographic variables were used: age of schoolchild in years (0 = 6-7 yrs.; 1 = 8–10 yrs.; 2 = 11-12 yrs.); sex of schoolchild (0 = female; 1 = male); head of household (0 = mother; 1 = father; 2 = other); mother's and father's ages in years (continuous format); mother's and father's education level (0 = primary; 1 = middle; 2 = high; 3 = Bachelor's or higher); health insurance coverage (0 = uninsured, 1 = IMSS/ISSSTE, 2 = PEMEX, SEDENA, SEMAR, 3 = private, 4 = Seguro Popular); and automobile in household (0 = yes; 1 = no). Four schoolchild oral health variables were measured: brushing frequency (0 = at least once a day; 1 = less than once a day); toothpaste use (0 = at least once a day; 1 = less than once a day); dental floss use (0 = at least once a week; 1 = never, do not know); and mouthwash use (0 = at least once a week; 1 = never, do not know). Parent/guardian variables included brushing frequency (0 = at least once a day; 1 = less than once a day) and perception of schoolchild's oral health condition (0 = bad/very bad; 1 = regular; 2 = good/very good).

Using a polychoric correlation as part of a principal components analysis, three different interrelated groups of variables were formed. The first group encompassed two variables indicating socioeconomic position, one referring to housing characteristics (e.g., wall, roof, and floor building materials, presence/absence of a kitchen, bathroom characteristics, and number of bedrooms) and the other to household appliances (e.g., refrigerator, stove, television, and telephone). A second group consisted of three variables addressing frequency of candy, fried foods, and fruit consumption. The third group was one variable on parent/guardian knowledge of schoolchild oral health. After generating the principal component for each of these six (continuous) variables, the socioeconomic condition indices were categorized into quartiles, while the food intake and oral health knowledge indices were categorized into tertiles. Depending on the variable, the first indicated the lowest level and the last the highest level.

### 2.3. Statistical Analysis

After cleaning the database, we performed a descriptive analysis of the studied variables, estimating frequencies and percentages for each category of qualitative variable. The quantitative variables were analyzed by calculating the mean and standard deviation (SD). In the bivariate analysis, contingency tables were generated for the dental pain dependent variable with each independent variable and the Pearson *χ*^2^-type test of independence run. Mann-Whitney *U* tests for independent samples were applied for mother's and father's age. These were fitted to a binary logistical regression multivariate model to estimate the strength of association between the dependent and independent variables. Results were expressed as an odds ratio (OR) with a 95% confidence interval (CI 95%). Statistical significance for *p* values was set at <0.05. Only those variables with *p* < 0.25 in the bivariate model were considered in the multivariate model. In response to correlation between groups (school variable cluster), confidence intervals were calculated with robust Huber-White standard deviations. This occurred because similarity was higher and therefore had greater correlation, among schoolchildren from the same school; that is, the clusters were distinctly different [23]. The model fit was evaluated with the Hosmer-Lemeshow statistic [24]. All statistical analyses were run with the Stata ver. 13® package.

### 2.4. Ethical Aspects

This methodology met study subject protection guidelines and relevant Helsinki ethical regulations. The study protocol was approved by the Ethics and Research Committee of the Autonomous University of the State of Hidalgo (Universidad Autónoma del Estado de Hidalgo [UAEH]) and the committees of the National Institute of Public Health (UAEH Institutional Ethical Review Committee code: UAEH-DI-ICSA-ODO-CF-016). Written consent was obtained from all the patients/guardians.

## 3. Results

### 3.1. Sample Characteristics

The 1,404 schoolchildren in the sample had a mean age of 8.96 ± 1.99 years and 49.9% were female (Tables 1 and 2). The participant's mothers had a mean age of 34.8 ± 6.1 years, and fathers' mean age was 37.7 ± 6.32 years. In 77.6% of the participating families, the father was head of the household. The largest proportion of mothers (36.8%) had completed some or all of middle school, while the largest proportion of fathers (32.5%) had completed some or all of high school. Most (51.8%) of the participating families had public sector health insurance, through either the IMSS* (Instituto Mexicano de Seguro Social)* or ISSSTE* (Instituto de Seguridad y Servicios Sociales de los Trabajadores del Estado)*. Most (85.7%) of the schoolchildren brushed their teeth at least once per day and always used toothpaste (90.9%). However, most had never used/did not know about dental floss (80.6%) or mouthwash (71.8%). Parent/guardian brushing frequency was largely “at least once per day” (89.4%), and the majority (45.2%) reported their child's oral health to be “regular.” Half (49.9%) of the schoolchildren were reported to have had dental pain at some time during the twelve months prior to the study.

### 3.2. Bivariate Analysis of Reported Pain versus Independent Variables

Of the half of the children who reported to have had dental pain, the largest proportion were male, affiliated with the Seguro Popular system, located in the lowest socioeconomic level in terms of housing characteristics, and lived in households without an automobile (Table 3). Unexpectedly, the highest dental pain prevalence was observed in the highest socioeconomic level based on domestic appliances.

Of the schoolchildren with reported dental pain, a larger proportion had high fried food intake and low fruit intake (Table 4). These children also had lower brushing frequency and toothpaste use values. An unexpected result was that those who used dental floss and mouthwash at least once a week had a higher dental pain frequency than those who did not use these hygiene tools. Dental pain was also more frequent among the schoolchildren with a parent/guardian who reported lower brushing frequency, had a regular or high oral health information level, and perceived their child's oral health as being bad/very bad or regular.

### 3.3. Logistical Regression Multivariate Model

Our multivariate model results revealed that, for each year of increase in mother's age, the possibility of dental pain in the schoolchild decreased (OR_A_ = 0.98; CI 95% = 0.96–0.99) (Table 5). Schoolchildren in the top quartile of housing characteristics (NSE) had a lower probability (OR_A_ = 0.98; CI 95% = 0.96–0.99) of experiencing dental pain than those in lower quartiles. Living in a home with no automobile increased the probability of experiencing dental pain by 49% compared to the children in homes with automobiles. In contrast to children with low reported fried food intake, those with a high intake had a 2.34% (CI 95% = 1.42–3.88) greater probability of dental pain, and those with moderate intake had an 87% (CI 95% = 1.30–2.69) greater probability. High (OR_A_ = 0.61; CI 95% = 0.42–0.88) and moderate (OR_A_ = 0.68; CI 95% = 0.48–0.97) fruit intake decreased the probability of experiencing dental pain compared to those with low intake. Schoolchildren who brushed their teeth less than once per day had 2.31 times greater probability of having dental pain than those who brushed at least once per day. A parent/guardian perception of good/very good (OR_A_ = 0.34 CI 95% = 0.19–0.62) and regular (OR_A_ = 0.39 CI 95% = 0.19–0.80) child oral health lowered the possibility of dental pain compared to a bad/very bad perception. Surprisingly, parent/guardian nonuse of mouthwash lowered (OR_A_ = 0.46; CI 95% = 0.27–0.78) child probability of having dental pain in contrast to those who used it at least once per week.

## 4. Discussion

To our knowledge, our study is one of the first to address the prevalence of and factors associated with dental pain in Mexico. The frequently observed relationship between dental pain and dental caries in this age group makes it an important variable. Identifying dental pain in schoolchildren can be a good indicator of the need for curative or emergency treatment, an estimator of the proportion of people who may use oral health services in the future, and therefore a key datum in planning oral health service needs.

Half (49.9%) of the studied schoolchildren had experienced dental pain in the twelve months prior to the survey, and different variables were associated with its presence. This prevalence is higher than the 35.7% reported for schoolchildren from private and public schools in Brazil [10, 25] but similar to the 47.6% reported for schoolchildren in Uganda [26]. Higher prevalences have been reported in people 10 years of age and older in Chad (64.1%) [27]; in eight-year-old children in Sri Lanka (49% [self-reported] to 53% [reported by parents]) [28]; and in eight- to ten-year-old children in Western Cape, South Africa, in the two months prior to the study (70%) [29]. These differences in dental pain prevalence can be attributed to various causes, including the development context in different countries and locations, disease level variations between the studied populations, health system response to a population's oral health needs, and the methodologies used in each study.

In a previous study on parent/guardian influence and responsibility for child health, parents acknowledged the benefits of brushing teeth with fluoride toothpaste. Even so, the parents of children that had experienced caries and dental pain believed that the causes were beyond their control, due to genetics, attributed to health problems intrinsic to childhood, or just random [30].

The positive impact of greater mother's age on dental pain may be due to the information and experience older mothers have accumulated throughout their lives. This coincides with a study in the United Kingdom indicating that greater mother's age favorably affects child cognitive, behavioral, and health condition [31].

Describing the association between an individual's pain and their socioeconomic level (based here on the socioeconomic variables remaining in the final model: housing characteristics and automobile in household) can be quite complex since socioeconomic position is a multifactorial construct [32]; however, a number of hypotheses have been reported to plausibly explain this association [33]. In school-age children, dental pain is largely of odontogenic origin. Addressing the causes of dental pain in Mexico can be problematic since the public health system offers only limited coverage for oral health treatments. The private sector is the only source of adequate treatment, although this requires substantial out-of-pocket expenditure.

Fried food intake was associated with a greater possibility of dental pain in the studied schoolchildren. A possible explanation for this is the greater presence of dentobacterial plaque caused by intake of fried, high carbohydrate foods between regular meals. This in turn can directly affect teeth and gums exposed for long periods [34]. In conjunction with poor oral health practices, this could explain the greater possibility of dental pain in this group of children. A number of studies document the effects of sweet and salty snacks on dental caries, one of the main causes of dental pain [35]. Mitigating the effect of poor health habits can be done by promoting greater physical activity, higher fruit and vegetable intake, and school breakfasts [36]. Although programs promoting healthy habits do exist in Mexico, schoolchildren are exposed to a number of nutritional risks, such as purchasing power, skipping a meal (usually breakfast), and junk food for sale near schools [37]. Indeed, the most prevalent diet among schoolchildren in Mexico is one of fruit, salty fried snacks, candy, and pastries [38]. In the present results, the schoolchildren who consumed fruit had a decreased probability of developing dental pain, which may be explained by a healthier lifestyle, including a lower intake of cariogenic foods [39]. This coincides with a study done in the United States indicating that people with more severe caries also had poor oral hygiene and consumed high levels of sugars and fats and low levels of fruits and vegetables [40]. Further research is needed in Mexico to better understand the relationship between lifestyle and oral health.

Brushing of teeth mechanically removes and disorganizes dental biofilm, limiting its ability to cause disease. It is the most cost effective dental care instruction, is widely recommended for maintaining oral health, and is a habit best inculcated at an early age [41]. In the present results, lower brushing frequency was associated with the presence of dental pain, which can be explained by a lower frequency of caries (and therefore dental pain) in those children with better oral hygiene habits. Mouthwash is normally seen as a positive addition to oral hygiene [42], but in the present results dental pain frequency was lower in the schoolchildren whose parents stated they did not use mouthwash or did not know about it; no explanation is immediately apparent for this result.

Health literacy is the “ability to obtain, process and understand information” and the services needed to make adequate health decisions [43]. Some studies have documented a relationship between health knowledge and clinical results [44], but few have addressed the association between health knowledge and oral health [43]. Parental attitudes can have direct repercussions on child oral health, as demonstrated in a study showing that limited oral health knowledge among parents was associated with negative attitudes towards oral health, low frequency of healthy behaviors, and worse oral health in children [44]. The present results, however, suggested an association between dental pain in schoolchildren and high to regular knowledge of oral health practices among parents. These parents may more readily take their child for dental treatment, which can cause discomfort identified by the child as dental pain, even though it is transitory and forms part of a dental therapeutic procedure.

Parents are vital to child development because they are responsible for seeking timely treatment for any disorders that might occur in the child. Oral health is a component of overall good health since it can affect taste, mastication, speech, and facial expression [45]. Identifying the factors that influence the association between parent perception and child oral health could help primary care givers and oral health service providers (e.g., health promotors, dentists) to understand why schoolchildren do not receive early attention for dental problems at home or with a general dentist. This is particularly important because this association often results in children requiring more invasive treatments [46]. Parent perceptions of their child's oral health condition as good/very good or regular were associated with a lower dental pain frequency than the most affected group. Perhaps clinical conditions (e.g., color and structure) and symptoms influence parent perception, motivating them to make the decision to seek preventative and/or curative care, thus anticipating problems such as dental caries [47].

The present study has three main limitations. The first is that, due to its cross-sectional design, the temporality between variables is not accurate (temporality ambiguity), potentially causing reversed directions, and representing a possible inherent bias. Information selection bias (i.e., memory) is the second limitation; dental pain was explored over a 12-month period prior to the survey, affecting memory accuracy, be it in the individual or in cooperation with the parent/guardian-child. The third limitation is that the study does not include all of the schoolchildren in the region, meaning it is not representative of the state's overall school population. Future research should now address the source(s) of dental pain in schoolchildren.

## 5. Conclusions

Overall, dental pain prevalence in the sampled population was high: 1 in every 2 children had experienced dental pain in the 12 months prior to the survey. Among the diverse factors affecting this prevalence, socioeconomic condition (housing characteristics and presence of an automobile in the home) suggested the presence of inequalities in oral health status and access to dental care among the studied children. Future studies should now evaluate the response of oral health services to people with dental pain.

## Figures and Tables

**Table 1:** Sociodemographic and socioeconomic data for schoolchildren aged 6 to 12 years in dental pain survey.

Variables	*n*	%
*Age*		
6-7 yrs.	409	29.1
8–10 yrs.	609	43.4
11-12 yrs.	386	27.5

*Sex*		
Female	701	49.9
Male	703	50.1

*Head of household*		
Mother	241	17.2
Father	1090	77.6
Other	73	5.2

*Mother's education level*		
Primary	137	9.8
Middle	517	36.8
High	493	35.1
Bachelor's or higher	257	18.3

*Father's education level*		
Primary	125	9.2
Middle	365	26.8
High	443	32.5
Bachelor's or higher	430	31.5

*Health insurance*		
Uninsured	433	30.8
IMSS/ISSSTE	727	51.8
PEMEX/SEDENA/SEMAR	68	4.8
Private	49	3.5
Seguro Popular	127	9.1

*SEL (household appliances)*		
1st quartile	351	25.0
2nd quartile	352	25.1
3rd quartile	351	25.0
4th quartile	350	24.9

*SEL (housing characteristics)*		
1st quartile	356	25.3
2nd quartile	354	25.2
3rd quartile	345	24.6
4th quartile	349	24.9

*Automobile in home*		
Yes	893	63.6
No	511	36.4

	*n*	*Mean* ± *sd*

*Mothers' age*	1404	34.89 ± 6.06
*Father's age*	1363	37.72 ± 6.32

**Table 2:** Risk indicator (diet, oral health habits, and behavior) distribution among schoolchildren aged 6 to 12 years in dental pain survey.

Variables	*n*	%
*High calorie food intake*		

Candies		
Low	470	33.5
Moderate	466	33.2
High	468	33.3

Fried food		
Low	471	33.5
Moderate	467	33.3
High	466	33.2

Fruit		
Low	469	33.4
Moderate	473	33.7
High	462	32.9

*Oral health habits*		

Brushing frequency		
At least once a day	1204	85.7
Less than once a day	200	14.3

Toothpaste use		
At least once a day	1276	90.9
Less than once a day	128	9.1

Dental floss use		
At least once a week	273	19.4
Never, do not know of it	1131	80.6

Mouthwash use		
At least once a week	396	28.2
Never, do not know of it	1008	71.8

*Parent oral health knowledge and habits*		

Brushing frequency		
At least once a day	1255	89.4
Less than once a day	149	10.6

Knowledge of oral health		
Sufficient	468	33.3
Regular	468	33.3
Insufficient	468	33.3

Perception of child's oral health		
Very bad/bad	158	11.3
Regular	635	45.2
Good/very good	611	43.5

**Table 3:** Bivariate analysis of sociodemographic and socioeconomic variables for schoolchildren aged 6 to 12 years in dental pain survey.

Variables	No pain *n* = 704 (50.1%)	Pain *n* = 700 (49.9%)	*p* value^*∗*^
*Age*			
6-7 yrs.	210 (51.3)	199 (48.7)	0.601
8–10 yrs.	296 (48.6)	313 (51.4)	
11-12 yrs.	198 (51.3)	188 (48.7)	

*Sex*			
Female	375 (53.5)	326 (46.5)	**0.012**
Male	329 (46.8)	374 (53.2)	

*Head of household*			
Mother	131 (54.4)	110 (45.6)	0.356
Father	537 (49.3)	553 (50.7)	
Other	36 (49.3)	37 (50.7)	

*Mother's education level*			
Primary	63 (46.0)	74 (54.0)	0.103
Middle	242 (46.8)	275 (53.2)	
High	260 (52.7)	233 (47.3)	
Bachelor's or higher	139 (54.1)	118 (45.9)	

*Father's education level*			
Primary	56 (44.8)	69 (55.2)	0.190
Middle	168 (46.0)	197 (54.0)	
High	225 (50.8)	218 (49.2)	
Bachelor's or higher	226 (52.6)	204 (47.4)	

*Health insurance*			
Uninsured	235 (54.3)	198 (45.7)	**0.010**
IMSS/ISSSTE	358 (49.2)	369 (50.8)	
PEMEX/SEDENA/SEMAR	39 (57.4)	29 (42.6)	
Private	25 (51.0)	24 (49.0)	
Seguro Popular	47 (37.0)	80 (63.0)	

*SEL (household appliances)*			
1st quartile	205 (58.4)	146 (41.6)	**0.004**
2nd quartile	170 (48.3)	182 (51.7)	
3rd quartile	170 (48.4)	181 (51.6)	
4th quartile	159 (45.4)	191 (54.6)	

*SEL (housing characteristics)*			
1st quartile	150 (42.1)	206 (57.9)	**<0.001**
2nd quartile	167 (47.2)	187 (52.8)	
3rd quartile	179 (51.9)	166 (48.1)	
4th quartile	208 (59.6)	141 (40.4)	

*Automobile in home*			
Yes	489 (54.8)	404 (45.2)	**<0.001**
No	215 (42.1)	296 (57.9)	

	*Mean ± sd*	*Mean ± sd*	

*Mothers' age*	35.47 ± 6.15	34.30 ± 5.91	**0.0001** ^*∗∗*^
*Fathers' age*	37.97 ± 6.36	37.48 ± 6.28	**0.0436** ^*∗∗*^

^*∗*^Pearson *χ*^2^ test; ^*∗∗*^Mann-Whitney test.

**Table 4:** Bivariate analysis of risk indicators (diet, oral health habits, and behavior) among schoolchildren aged 6 to 12 years in dental pain survey.

Factors	No pain *n* (%)	Pain *n* (%)	*p* value^*∗*^
*High calorie food intake*			

Candies			
Low	230 (48.9)	240 (51.1)	0.502
Moderate	244 (52.4)	222 (47.6)	
High	230 (49.2)	238 (50.8)	

Fried food			
Low	268 (56.9)	203 (43.1)	**<0.001**
Moderate	230 (49.2)	237 (50.8)	
High	206 (44.2)	260 (55.8)	

Fruit			
Low	207 (44.1)	262 (55.9)	**0.005**
Moderate	247 (52.2)	226 (47.8)	
High	250 (54.1)	212 (45.9)	

*Oral health habits*			

Brushing frequency			
At least once a day	640 (53.2)	564 (46.8)	**<0.001**
Less than once a day	64 (32.0)	136 (68.0)	

Toothpaste use			
At least once a day	662 (51.9)	614 (48.1)	**<0.001**
Less than once a day	42 (32.8)	86 (67.2)	

Dental floss use			
At least once a week	121 (44.3)	152 (55.7)	**0.032**
Never, do not know of it	583 (51.5)	548 (48.5)	

Mouthwash use			
At least once a week	157 (39.7)	239 (60.3)	**<0.001**
Never, do not know of it	547 (54.3)	461 (45.7)	

*Parent oral health knowledge and habits*			

Brushing frequency			
At least once a day	656 (52.3)	599 (47.7)	**<0.001**
Less than once a day	48 (32.2)	101 (67.8)	

Knowledge of child's oral health			
Sufficient	285 (60.9)	183 (39.1)	**<0.001**
Regular	218 (46.6)	250 (53.4)	
Insufficient	201 (42.9)	267 (57.1)	

Perception of child's oral health			
Very bad/bad	46 (29.1)	112 (70.9)	**<0.001**
Regular	325 (51.2)	310 (48.8)	
Good/very good	333 (54.5)	278 (45.5)	

^*∗*^Pearson *χ*^2^ test.

**Table 5:** Multivariate analysis of dental pain in schoolchildren aged 6 to 12 years in dental pain survey.

Variables	OR	CI 95%	*p* value
*Mother's age*	0.98	0.96–0.99	**0.019**

*NSE (housing characteristics)*			
1st to 3rd quartile (−)	1^*∗*^		
4th quartile (+)	0.52	0.30–0.92	**0.026**

*Automobile in home*			
Yes	1^*∗*^		
No	1.49	1.14–1.93	**0.003**

*Fried food intake*			
Low	1^*∗*^		
Moderate	1.87	1.30–2.69	**0.001**
High	2.34	1.42–3.88	**0.001**

*Fruit intake*			
Low	1^*∗*^		
Moderate	0.68	0.48–0.97	**0.033**
High	0.61	0.42–0.88	**0.009**

*Brushing frequency *			
At least once a day	1^*∗*^		
Less than once a day	2.31	1.51–3.53	**0.000**

*Mouthwash use*			
At least once a week	1^*∗*^		
Never, do not know of it	0.46	0.27–0.78	**0.004**

*Knowledge of child's oral health*			
Sufficient	1^*∗*^		
Regular	2.09	1.43–3.04	**0.000**
Insufficient	2.72	1.55–4.77	**0.000**

*Perception of child's oral health*			
Very bad/bad	1^*∗*^		
Regular	0.39	0.19–0.80	**0.011**
Good/very good	0.34	0.19–0.62	**0.000**

Note: Model fitted to variables in table; ^*∗*^reference category; model fit: Hosmer-Lemeshow *χ*^2^(8) = 9.42; *p* = 0.3081.
